# Social and Structural Determinants of Health Associated with COVID-19 Vaccine Hesitancy among Older Adults in the United States

**DOI:** 10.3390/vaccines12050521

**Published:** 2024-05-10

**Authors:** Kingsley Kalu, Gulzar Shah, Ho-Jui Tung, Helen W. Bland

**Affiliations:** Jian-Ping Hsu College of Public Health, Georgia Southern University, Statesboro, GA 30458, USA; kk13870@georgiasouthern.edu (K.K.); htung@georgiasouthern.edu (H.-J.T.); hwbland@georgiasouthern.edu (H.W.B.)

**Keywords:** vaccine hesitancy, immunocompromised nature, social determinants of health, structural determinants of health, religion, vaccine-preventable disease, older adults

## Abstract

State-level COVID-19 vaccination rates among older adults have been uneven in the United States. Due to the immunocompromised nature of older adults, vaccine hesitancy increases the risk of morbidity and mortality. This study aims to determine the association between the social determinants of health, the structural determinants of health, and COVID-19 vaccine hesitancy among older adults in the United States. Secondary data from the Health and Retirement Study (HRS) dataset were used. A descriptive analysis and multinomial multivariable logistic regression were performed to examine the association of the independent variables—gender, age, race, immigration status, marital status, broadband internet access, social security income, Medicare coverage, education, and frequency of religious service—with the dependent variable, vaccine hesitancy. Compared to the respondents with no vaccine hesitancy and without the specific predictor, the respondents who reported religious attendance at least once/week were more likely to be “somewhat hesitant”, divorced respondents had higher odds of being “somewhat hesitant”, and older adults aged 65–74 years were more likely to be “very hesitant” or “somewhat hesitant” about the COVID-19 vaccine. Compared to the respondents with no vaccine hesitancy and without the specific predictor, females had higher odds of being “very hesitant”, “somewhat hesitant”, or a “little hesitant”, and African Americans were more likely to be “very hesitant”, “somewhat hesitant”, or a “little hesitant” about the COVID-19 vaccine. Addressing these factors may limit the barriers to vaccine uptake reported among older adults and improve herd immunity among the immunocompromised population.

## 1. Introduction

Vaccination is considered the most significant achievement in public health since the dawn of the 18th century because it has significantly contributed to the reduction in vaccine-preventable infectious diseases [1,2]. From the discovery of smallpox vaccination to the present, a growing proportion of people has shown reluctance towards vaccinations, leading to numerous anti-vaccine campaigns and activities [3,4,5].

Vaccine hesitancy is a set of beliefs and behaviors exhibited to reject vaccination despite vaccine availability [6,7]. The Sage Working Group (WG) on vaccine hesitancy defined vaccine hesitancy “as the delay in acceptance or refusal of vaccination despite the availability of vaccination services”. “Vaccine hesitancy is complex and context-specific, varying across time, place, and vaccines, which is influenced by factors such as complacency, convenience, and confidence” [8,9]. Vaccine hesitancy occurs across a behavioral continuum, because some vaccine-hesitant individuals completely accept vaccines without hesitation, whereas others refuse them entirely without hesitation, yet others are between these two extremes [9,10]. Thus, the hesitancy continuum is sometimes measured as refusing all vaccines, refusing but unsure, refusing some vaccines, delaying, accepting some, accepting but unsure, and accepting all vaccines [9].

Vaccine hesitancy is a critical public health challenge in the fight against preventable infectious diseases. It has been a problem for global health since the discovery of vaccination [11,12,13]. Vaccine hesitancy makes it difficult to achieve herd immunity, which has led to an increased risk of mortality and morbidity in immunocompromised people, such as pregnant women, older adults, and children within the community [14,15,16]. Additionally, vaccine hesitancy can lead to increased healthcare expenditure, adverse health outcomes, and more burden on families, the healthcare system, and the government [17,18].

As of 2021, the United States had around 55.7 million adults who were aged 65 years or more. The majority of these older Americans were women with at least one chronic disease [19]. Older adults have an increased risk of adverse health outcomes when an infection occurs due to their limited regenerative capacity, their immunocompromised nature, and the presence of co-morbidities [20].

During the COVID-19 pandemic in the United States, there were uneven COVID-19 vaccination rates among older adults, with the lowest rate being in Utah (58%) [21]. Between 2020 and 2023, approximately 868,831 COVID-19-related deaths occurred in the US among adults aged 65 years and older, with men accounting for 53% of the deaths [22]. Although there was a decline in COVID-19 mortality among older adults during the rollout of vaccination in December 2020 in the United States, COVID-19 deaths among adults 65 years and older were reported to have increased to about 88% in September 2022 [23]. During the prevalence of Omicron BA.2 and Delta variants in 2022, adults aged 65 years and above in America experienced higher COVID-19 hospitalization rates compared to younger adults [18].

The Advisory Committee on Immunization Practices (ACIP) recommended the bivalent COVID-19 vaccine dose in September 2022. Despite that recommendation, 76% of US adults aged 65 years and older who were hospitalized for COVID-19 had not received the bivalent dose, and 16% had not received any COVID-19 vaccine [24]. In 2023, older adults comprised nearly 90% of COVID-19-related deaths in the United States. They make up 63% of all COVID-related hospitalizations, with most of them having multiple comorbidities; only 24% had received the recommended COVID-19 bivalent vaccine [24,25]. The COVID-19 pandemic has led to adverse health outcomes, which can cause a significant healthcare burden [26]. The older adults who declined vaccination encountered increased healthcare burden and unfavorable health outcomes, such as morbidity, increased hospitalization, and significant COVID-19 death rates, because they tended to have immunocompromised status and comorbidities [18,23,27].

Social determinants of health (SDoHs) are the “conditions in which people are born, grow, work, live and the wider set of forces and systems shaping the conditions of daily life” [28]. Five domains reflect the SDoHs: (a) economic, e.g., income; (b) healthcare access and quality, and health insurance; (c) social and community context, e.g., marital status, neighborhood; (d) built environment, e.g., internet access; and (e) education access and quality [29]. Social Security income (SSI) serves as a foundation for income support and economic security programs to ease the burden of older adults in the United States [30]. Although the social security income benefit program may seem insufficient, it has been linked to socioeconomic health disparities [31]. Marital status as a determinant of health has been linked to health outcomes and factors in spouses providing cognitive, social, and emotional support and social integration within the community [32,33]. About 22 million American older adults (i.e., African American and Latino seniors) do not have and cannot access broadband internet at home, which is a significant determinant of health [34,35]. In addition, older adults without internet access lack access to healthcare and health-related information [34,36]. Education as a determinant of health has been linked to health outcomes, especially for older adults because it allows them to comprehend complex health information and enhance their health literacy [29]. Although the COVID-19 vaccines were freely administered regardless of insurance status in the United States [37], individuals with health insurance had the opportunity to inquire about health information from their primary healthcare provider [38]. It is pertinent to assess the impact of this health information, especially among older adults with health insurance coverage, because people were reluctant to take the COVID-19 vaccine when it became available due to the increasing source of misinformation [39].

Structural determinants of health (StrDoHs) are cultural, political, social, and economic structures that form the distribution of symbolic power, materials, and resources [40]. StrDoHs are the basis of health inequities, and they look at the interplay between socio-political factors because they examine the quality of the social determinants of health experienced by people in their neighborhoods and communities [40,41]. Although religion is considered a social and structural determinant of health, the latter can influence institutional and socioeconomic conditions, such as economic decisions, political parties, policy, racism, colonialism, and admittance to electoral offices [42]. Religion has also been linked to individual health outcomes via social support [43,44,45]. The five dimensions of religiosity are personal practice, religious exclusivity, religious belief, external practice (i.e., religious service attendance, social activities, and group membership), and religious salience [46]. Over 90% of American older adults consider themselves to be religious or spiritual. This could be attributed to the fact that around 50% of them attend religious services on a weekly basis, as well as engage in private religious practices, such as praying [47]. Few studies have tried to assess various concepts of religiosity and vaccine hesitancy; a study focused on religious identity being associated with COVID-19 vaccine intention [48]. Another study revealed that religious beliefs impact scientific and medically sound evidence, leading to vaccine hesitancy [49]. Martens and Rutjens (2022) showed that religiosity and spirituality contributed to ongoing COVID-19 vaccination rates [50]. Another study accessed common religious beliefs associated with vaccine hesitancy and its consequences [51]. Only a few studies have researched the frequency of religious service attendance and COVID-19 vaccine hesitancy in older adults in the United States.

Multiple studies have identified various factors that contribute to vaccine hesitancy. These include ethnicity, socioeconomic status, distrust, political affiliation, misinformation, and a culturally insensitive healthcare system as causes of vaccine hesitancy. These factors impact health disparities and are associated with vaccine intention [52,53,54,55,56,57]. A few studies have researched COVID-19 vaccine hesitancy and older adults using state-level data. A study looked at the impact of health equity in COVID-19 vaccination among older adults, focusing on occupation, language, and housing [58]. Another study examined the association between health information consumption, trust dynamics, and COVID-19 vaccine hesitancy among older adults [59]. In addition, a study focused on the determinants of vaccine acceptability in older adults aged 50 years and older by examining human immunodeficiency virus (HIV) disease, demographic characteristics, and psychosocial factors [60]. Cimone and colleagues evaluated the association between COVID-19 vaccine intention and perception in older adults of an integrated health system during June 2021–February 2021 [61]. In contrast, Sun and Rhubart focused on the association of rural-urban differences and disability and aging services and COVID-19 vaccination rates among older adults using county-level data [62]. Yet, another research study focused on the social and structural determinants of health to investigate attitudes and knowledge toward COVID-19 vaccine uptake among diverse racial and ethnic groups [54].

The current study is unique because it focuses on COVID-19 vaccine hesitancy among older adults before the onset and during the COVID-19 vaccine administration, and this aligns with the definition of vaccine hesitancy as a behavioral continuum. Limited studies have been conducted to identify the association between each domain of the SDoHs and vaccine hesitancy among older adults in the United States. In addition, none of the studies used a nationally representative sample of older adults in the United States (U.S.) to examine the association between the social and structural determinants of health and vaccine hesitancy in older adults in the United States. Lastly, this study aimed to assess the association between the social-structural determinants of health and vaccine hesitancy in older adults in the United States. These explanatory variables provide a new focus as to why vaccine hesitancy persists among older adults. The object of this study was to determine: (a) the association between the social determinants of health and vaccine hesitancy, and (b) the association between the structural determinants of health and vaccine hesitancy among older adults in the United States.

## 2. Materials and Methods

### 2.1. Data Source

The research used secondary data from the Health and Retirement Study (HRS), a nationally representative sample of the older adult population in the United States [63]. The National Institute on Aging (NIA), under the direction of the United States Congress, created the Health and Retirement Study to inform discussion at a national level about health and retirement issues among the growing population of older Americans. In 1992, the HRS launched a longitudinal survey of American older adults with a complex panel structure and sample design, which included information regarding successful aging (i.e., cognitive, public, and psychological); detailed health and economic information, behavior, and choices (i.e., health behaviors, work, and residence); and events and transitions (widowhood and institutionalization). The HRS participants represent all the United States population aged 50 years and older and are followed to death. The HRS participants are grouped in cohorts based on the year of birth. Every two years, new cohorts of participants are added, and the samples are refreshed with younger cohorts every six years. Although the HRS sample size ranges from 18,000 to 23,000 in any given wave [64,65,66], the sample size for this study was *N* = 2311. This was based on the dependent variable of interest and restricted to participants aged 65 years and older who answered the COVID-19 vaccine hesitancy question. The National Institute on Aging (NIA) classifies older adults in the United States as people aged 65 years or older, and this selected age group also represents the age of Medicare eligibility [67,68].

### 2.2. Sampling Design

A multi-stage probability sampling design of United States households involving geographical stratification, clustering, and oversampling of certain demographic groups was used for the HRS study [59,69]. This multi-stage area probability design consists of four stage selections the primary stage selection details information regarding core samples, Hispanic supplements, Black supplements, and Florida oversampling; the secondary stage selection of area details information of second-stage sampling unit (SSU) stratification, selection, and allocation; the third stage focuses on the selection of housing units-located geographical area; and the fourth stage details participant selection, in which the interviewer made a list of all household members within each sampled housing unit [70].

### 2.3. Data Collection

The COVID-19-related questions were incorporated in the 2020 HRS study, and the COVID-19 core interview data collection period was initially from March 2020 to June 2021 and repeated in 2022 [71]. The COVID-19 Project of the 2020 Health and Retirement Study (HRS) was administered to the 50% random sub-sample of households initially assigned to enhanced face-to-face interviewing (EFTF). Interviews were conducted via the web or telephone due to restrictions concerning social distancing and social contacts. Respondents who preferred in-person interviews were sent the self-administered leave-behind questionnaire [72,73,74]. Information on the questionnaire, data collection instruments, HRS COVID-19 data resources and release date, validation, and its application can be found at this link: https://hrs.isr.umich.edu/data-products/covid-19 (accessed on 10 February 2024)

### 2.4. Variables

#### 2.4.1. Dependent Variable

Although the data collection for the 2020 COVID-19 core Section started before the COVID-19 vaccine was available, the survey question was later updated to cover participants who did not take the vaccine when it became available. The survey question that operationalized vaccine hesitancy before the vaccine became available was “It’s possible there will be a vaccine for coronavirus in the next several months; how likely would you be to take a vaccine if it were available to you like a flu shot?” After the vaccine was made available, the participants who answered no to the question “Vaccines for the coronavirus have recently become available for some people. Have you received a vaccination shot for the coronavirus?” were then asked the update question “How likely are you to take a coronavirus vaccine when it becomes available to you?” The response categories both before and after the vaccine remained the same: very likely, somewhat likely, not very likely, and not at all likely. These were reverse-recoded into not at all hesitant, a little hesitant, somewhat hesitant, and very hesitant.

#### 2.4.2. Independent Variables

The independent variables reflecting the social determinants of health were measured as social security income, marital status, broadband internet access, educational level, and Medicare insurance coverage. The independent variable reflecting the structural determinant of health was measured as religiosity (i.e., frequency of religious attendance).

Social Security income: The social security income variable was measured using the survey question, “Do you currently receive any income from Social Security?” The response categories were Yes or No.

Marital status: The variable “marital status” was measured using the survey question “marital status” with the response categories of married, separated/divorced, widowed, never married, and marital status unknown. The original response categories were coded married, separated/divorced, widowed, and never married.

Internet access: The variable “Internet access” was measured using the survey question, “Do you regularly use the Internet (World Wide Web) for sending and receiving e-mail or for any other purpose, such as making purchases, searching for information, or making travel reservations?” The response categories were Yes or No.

Education: The variable “education” was measured using the survey question “Highest level of education?” The responses were No degree, GED, High school diploma, two-year college degrees, four-year college degrees, Master’s degree, professional degree, and degree unknown. The original response categories were recoded as high school diploma, unknown degree, college degree, graduate degree, and No degree.

Health insurance: The health insurance coverage variable was measured using the survey question, “Are you currently covered by Medicare health insurance?” The response categories were Yes or No. Religiosity: Capturing the structural determinant of health, the religiosity variable was measured using the survey question “How often do you attend religious service?” and the original response categories were “more than once a week, once a week, two or three times a month, one or more times a year, not at all”. The original responses were at least once a week, at least once a month, at least once a year, and not at all.

#### 2.4.3. Demographic Variables

The demographic variables of interest were age (65–74 years, 75–84 years, and 85 years and older), gender (Female and Male), race (African American, White/Caucasian, Other), and Immigration status (U.S.-born and Immigrant).

### 2.5. Analytical Methods

The data analysis consisted of descriptive and multivariable multinomial logistic regression. A descriptive analysis, such as mean, percentages, and frequencies, was conducted to describe the characteristics of the study population. A multinomial multivariable logistic regression was conducted to examine the association of the social determinants of health—marital status, broadband internet access, education, social security income, and health insurance—and demographic markers—age, gender, race, and immigration status with COVID-19 vaccine hesitancy.

Furthermore, a multinomial multivariable logistic regression was conducted to assess the association between the structural determinants of health—frequency of religious attendance—and demographic markers—age, gender, race, and immigration status—with COVID-19 vaccine hesitancy. Lastly, a multinomial multivariable logistic regression was conducted to assess the association of the social determinants of health—marital status, broadband internet access, education, social security income, and health insurance— and structural determinants of health—frequency of religious attendance—and demographic markers—age, gender, race, and immigration status—with COVID-19 vaccine hesitancy. The analysis was restricted to respondents aged 65 years and older who responded to the vaccine hesitancy question. Based on the dataset owner’s feedback on using sampling weights for a cross-sectional analysis, weights were not applied to the analysis. The IBM SPSS statistical software (version 29) was used for the analysis, and the statistical significance threshold was *p* ≤ 0.05.

## 3. Results

### 3.1. Unweighted Descriptive Studies and Characteristics of the Respondents

Table 1 shows the characteristics of the respondents and the descriptive statistics of the dependent and independent variables.

#### Characteristics of the Respondents

The study results (Table 1) reveal that more than half of the respondents were female (60%), and a large percentage identified their race as White (71%). The majority of the respondents were born in the United States (85%). The respondents within the 65–74 years age group were at least 53%, the 75–84 years age group was 34%, and the 85 years and older age group corresponded to 13% of the respondents. The married respondents were 48%, and 27% of the respondents were widowed. Forty-six percent of respondents had at least a high school degree. Thirty-seven percent of the respondents reported religious attendance at least once a week, and the same percentage reported no religious attendance. Close to 49% of the respondents were not hesitant to take the COVID-19 vaccine when available, and 26% were a little hesitant. And many respondents had Medicare coverage (93%) and social security income (91%).

Table 2 shows that, when compared to the respondents who were 85 years and older, those in the age group 65–74 years had higher odds of being “very hesitant” (AOR = 1.76, CI = 1.12–2.77) about the COVID-19 vaccine or “somewhat hesitant” (AOR = 2.04, CI = 1.19–3.49), rather than not being vaccine-hesitant. Compared to men, women were more likely to be “very hesitant” (AOR = 1.90, CI = 1.42–2.55) about the COVID-19 vaccine, “somewhat hesitant” (AOR = 1.95, CI = 1.42–2.67), or a “little hesitant” (AOR = 1.69, CI = 1.35–2.12), rather than not being vaccine-hesitant. Compared to White individuals, African Americans had higher odds of being “very hesitant” (AOR = 2.54, CI = 1.83–3.52) about the COVID-19 vaccine, “somewhat hesitant” (AOR = 2.36, CI = 1.66–3.36), or a “little hesitant” (AOR = 1.89, CI = 1.42–2.50) rather than not being vaccine-hesitant. Compared to the respondents who reported no religious attendance, the respondents who reported religious attendance at least once a week were more likely to be “somewhat hesitant” (AOR = 1.82, CI = 1.30–2.56) about the COVID-19 vaccine rather than not being vaccine-hesitant. Compared to the respondents with no degree, high school respondents had lower odds of being a “little hesitant” (AOR = 0.70, CI = 0.52–0.95) about the COVID-19 vaccine, and college degree respondents had lower odds of being a “little hesitant” (AOR = 0.60, CI = 0.42–0.85). Graduate respondents had lower odds of being “very hesitant” (AOR = 0.39, CI = 0.21–0.72) about the COVID-19 vaccine, “somewhat hesitant” (AOR = 0.38, CI = 0.20–0.72), or a “little hesitant” (AOR = 0.49, CI = 0.32–0.76) rather than being “very hesitant”. Compared to the unmarried respondents, the separated/divorced respondents were more likely to be “somewhat hesitant” (AOR = 2.32, CI = 1.15–4.68) about the COVID-19 vaccine rather than not being vaccine-hesitant. However, there was no association between social security income, immigration status, broadband internet use, Medicare coverage, and COVID-19 vaccine hesitancy among older adults.

## 4. Discussion

This study provides new findings that suggest that the frequency of religious attendance, marital status, and being 65–74 years old are associated with COVID-19 vaccine hesitancy in the United States. The authors found that increased frequencies of religious attendance were associated with higher odds of being vaccine-hesitant. Religiosity can significantly impact vaccine hesitancy because of a complex web of intertwined mediators and moderators between these two phenomena. For instance, religious groups differ in their beliefs concerning the relative role of science versus divinity in the prevention and cure of disease [75], which can result in variation in vaccine desirability and acceptability, as evident from religion-based vaccine exemptions [76]. People with different religious beliefs also vary in their contribution and receptivity to misinformation about the vaccines’ safety as well as their trust in medical professionals’ advice compared to such advice from their religious leaders [77].

Numerous studies have examined various forms of religiosity (e.g., the role of religiosity and prayer frequency) and their impact on vaccine hesitancy [49,51]. In the regression model that combined both the SDoH and StrDoH variables, the severity of hesitancy associated with religiosity reduced with participants who attended religious activity at least once a week having an almost two-fold increase in the odds of being somewhat hesitant about the COVID-19 vaccine. This level of association seen in the StrDoHs may be related to the refusal to validate governmental regulations, such as social distancing and institutional conflicts between religion, political affiliation, and science, especially regarding vaccination [78,79]. The variation of hesitancy may also be related to policies intervening on specific social determinants of health predictors and having influence mediated by existing religious practices, norms, and culture [40,44]. Public health organizations can partner with faith-based organizations to conduct health programs and use their “voice” to promote vaccine acceptance.

Also, the odds of vaccine hesitancy were higher for those with a marital status of separation and aged from 65 to 74 years.

This study showed that separated/divorced respondents among older adults were two times more likely to be “somewhat hesitant” about the COVID-19 vaccine. This could be due to a variety of factors, including emotional stress, changes in social support networks, or differences in health behavior following a separation or divorce. For divorced or separated individuals, the lack of a partner may result in less encouragement or support for vaccination, leading to higher rates of hesitancy [32,33]. In contrast, other studies showed no association between marital status and COVID-19 vaccine hesitancy [80]. This suggests the importance of social support and social ties and how they impact health behavior [81]. Groups targeted at separated or divorced people can be organized to provide social support and health information to the members.

The findings that older adults are hesitant to use the COVID-19 vaccine were consistent with previous studies [39,82]. However, it showed that older adults aged 65–74 years were about two times more likely to be “very hesitant” and “somewhat hesitant”. This could be due to a history of previous hesitancy, distrust in government, misinformation, and vaccine brands influencing vaccine intentions during the pandemic [59,83,84,85]. The results may suggest a worsening case of vaccine hesitancy since one would expect that age group to be more receptive than older adults. There is a need to explore how this misinformation and perception can be corrected. This study found that a higher level of education was negatively associated with vaccine hesitancy among older adults in the United States. The findings are consistent with other studies that found older respondents with a high school degree and above had higher odds of being COVID-19 vaccine-hesitant [86,87]. However, this study showed that older respondents with a high school degree were associated with being a little hesitant, college degree holders were associated with being very hesitant or little hesitant, and graduate degree holders were associated with being very hesitant, somewhat hesitant, and a little hesitant about the COVID-19 vaccine. This suggests that informed respondents were more knowledgeable about their health and willing to adopt preventive health services [86,88]. Policies that encourage access to quality education must be encouraged, and barriers to increasing levels of education must be eliminated.

The authors found no association between Medicare coverage and COVID-19 vaccine hesitancy. This is consistent with research evidence that health insurance coverage did not influence vaccine intention due to the freely administered COVID-19 vaccination [89]. The research study also found no association between social security income and COVID-19 vaccine hesitancy, and this may suggest that it does not influence vaccine decision-making in older adults, even though social security income could improve health outcomes and well-being [90]. Although past studies showed that access to broadband internet impacted vaccine intention [8,91], this study found no association between internet access and COVID-19 vaccine hesitancy among older adults. The current study also found no association between immigrant status and COVID-19 vaccine hesitancy, showing an assimilation with the native population concerning vaccine acceptance.

The results show higher odds of vaccine hesitancy among females and African Americans. Although this study is consistent with previous studies suggesting increased vaccine hesitancy among women than men [92,93], this study found that older female respondents had a two-fold increase in hesitation about the COVID-19 vaccine. The fear of possible vaccine side effects, especially since more women are their family’s caregivers, may suggest their level of hesitancy [94]. Women ambassadors can be empowered with health information to take it to their families and communities. The results of this research study agree with those of other studies that showed African Americans were more likely to be vaccine-hesitant [80]. However, this study showed that African Americans had about a three-fold increase in being “very hesitant” about the COVID-19 vaccine and a two-fold increase in being “somewhat hesitant” or a “little hesitant”. This suggests a long history of hesitancy toward vaccination, racism, healthcare, and biomedical mistrust [80,95]. Despite past efforts to gain the trust of African Americans in the health system, it appears that not much progress has been made. Public health stakeholders need to engage African American influencers who can help to advocate the benefits of vaccination. Engaging this racial group through community-based participatory research may help to increase confidence since they are part of the process from vaccine development to roll-out.

This study’s findings should be viewed in the context of its limitations. First, due to the study design, our study cannot establish causality between the variables of interest. Also, the use of secondary data limits the variables available for analysis. For instance, only one dimension of religiosity (i.e., frequency of religious attendance) for the structural determinants of health was considered. This study had smaller analytic samples of the 2020 COVID-19 HRS data than full samples of HRS, which created a potential for selection bias due to the number of missing variables. The study findings are still reasonably robust and highly useful, given its strength of using a nationally representative dataset to analyze the association between the variables of interest. This study contributes to a critical body of knowledge to support health equity-related public health practice and policy initiatives on vaccine hesitancy as it focuses on older adults, often a forgotten vulnerable population.

## 5. Conclusions

Our study found that social and structural determinants of health were associated with vaccine hesitancy among older adults in the United States. Addressing social and structural determinants of vaccine hesitancy upstream will reduce the downstream disparities in vaccine uptake. Vaccine acceptance will improve herd immunity, mainly benefiting the immunocompromised population. This study provides valuable quantitative empirical evidence to guide policy and public health practice addressing vaccine hesitancy. Future studies conducting qualitative research on vaccine hesitancy will provide additional insights into and context for interventions and initiatives vested in improving vaccine uptake.

## Figures and Tables

**Table 1:** Descriptive statistics of the study participants’ characteristics, 2020–2022.

Variables	Frequency	Percentages
Dependent Variable		
Vaccine Intention		
Not Hesitant	1142	49
Little Hesitant	590	26
Somewhat Hesitant	259	11
Very Hesitant	320	14
Independent Variables		
Older Adults		
65–74 years	1234	53
75–84 years	777	34
85 years and older	300	13
Immigration Status		
US-born	1968	85
Immigrant	343	15
Gender		
Female	1405	61
Male	906	39
Ethnicity/Race		
Black	464	20
Other	198	9
White	1644	71
SDoHs Educational Level		
High School	1080	47
Unknown Degree	30	1
College Degree	484	21
Graduate Degree	256	11
No Degree	461	20
Marital Status		
Married	1105	48
Separated/Divorce	433	19
Widowed	624	27
Never Married	139	6
Social Security Income		
Yes	2080	91
No	200	9
Broadband Internet Access		
Yes	1334	59
No	937	41
Medicare Health Insurance		
Yes	2117	93
No	162	7
StrDoHs Frequency of religious attendance		
At least once a week	836	37
2/3 times a week	206	9
At least one/more times a month	375	17
Not at all	837	37

Abbreviations: SDoHs, Social determinants of health; StrDoHs, Structural determinants of health. Note. Total N = 2311.

**Table 2:** Multinomial multivariable logistic regression model showing the association between the socio-structural and demographic determinants of health and COVID-19 vaccine hesitancy among older adults.

	Very Hesitant				Somewhat Hesitant				Little Hesitant			
		95% CI				95% CI				95% CI		
	AOR	UL	LL	Sig	AOR	UL	LL	Sig	AOR	UL	LL	Sig
Marital Status												
Never Married	(Ref. Category)				(Ref. Category)				(Ref. Category)			
Married	0.99	0.57	1.72	0.96	1.52	0.77	3.00	0.22	1.20	0.76	1.89	0.43
Separated/divorced	1.34	0.74	2.40	0.34	**2.32**	1.15	4.68	0.02	1.06	0.65	1.75	0.81
Widowed	1.30	0.73	2.31	0.38	1.47	0.73	3.00	0.28	1.02	0.63	1.65	0.95
Immigration Status												
Immigrant	(Ref. Category)				(Ref. Category)				(Ref. Category)			
US-born	1.13	0.75	1.72	0.56	1.13	0.71	1.80	0.60	1.21	0.86	1.71	0.27
Educational Level												
No degree	(Ref. Category)				(Ref. Category)				(Ref. Category)			
High school	0.91	0.63	1.31	0.61	0.75	0.50	1.13	0.18	**0.70**	0.52	0.95	0.02
Unknown degree	1.44	0.53	3.92	0.48	0.62	0.16	2.33	0.48	0.48	0.16	1.40	0.18
College	0.65	0.41	1.02	0.06	0.73	0.45	1.17	0.19	**0.60**	0.42	0.85	0.005
Graduate	**0.39**	0.21	0.72	0.003	**0.38**	0.20	0.72	0.003	**0.49**	0.32	0.76	0.001
Social Security Benefits												
No social security income	(Ref. Category)				(Ref. Category)				(Ref. Category)			
Social security income	1.09	0.65	1.82	0.75	0.94	0.54	1.62	0.82	0.89	0.60	1.34	0.58
Health Insurance												
No medical health insurance	(Ref. Category)				(Ref. Category)				(Ref. Category)			
Presence of health insurance	0.91	0.53	1.56	0.72	1.43	0.75	2.72	0.28	1.25	0.78	2.00	0.36
Broadband Internet Use												
No internet use	(Ref. Category)				(Ref. Category)				(Ref. Category)			
Internet use	0.76	0.56	1.03	0.08	1.11	0.79	1.55	0.56	1.02	0.80	1.31	0.87
Religiosity												
Frequency of religious attendance	(Ref. Category)				(Ref. Category)				(Ref. Category)			
At least once a week	1.29	0.95	1.75	0.10	**1.82**	1.30	2.56	<0.001	1.27	0.99	1.63	0.06
2/3 times a month	0.77	0.47	1.28	0.32	0.84	0.47	1.50	0.55	1.25	0.86	1.82	0.23
At least one/more times a month	0.79	0.53	1.20	0.27	1.18	0.77	1.82	0.46	0.99	0.73	1.35	0.95
Age												
85 years and older	(Ref. Category)				(Ref. Category)				(Ref. Category)			
65–74 years	**1.76**	1.12	2.77	0.02	**2.04**	1.19	3.49	0.01	1.34	0.93	1.93	0.11
75–84 years	0.89	0.57	1.40	0.62	1.26	0.73	2.15	0.41	1.12	0.79	1.59	0.53
Gender												
Male	(Ref. Category)				(Ref. Category)				(Ref. Category)			
Female	**1.90**	1.42	2.55	<0.001	**1.95**	1.42	2.67	<0.001	**1.69**	1.35	2.12	<0.001
Race												
White	(Ref. Category)				(Ref. Category)				(Ref. Category)			
Black	**2.54**	1.83	3.53	<0.001	**2.36**	1.66	3.36	<0.001	**1.89**	1.42	2.50	<0.001
Other	1.26	0.76	2.07	0.37	1.09	0.62	1.91	0.78	0.80	0.52	1.24	0.32
The reference outcome category is Not Hesitant												

Note. AOR, Adjusted odds ratio; CI, Confidence interval; Ref. Category, Reference category; LL, Lower limit; UL, Upper limit; Sig, Significant level at *p* ≤ 0.05.

## Data Availability

The dataset is publicly available at https://hrs.isr.umich.edu/data-products (accessed on 10 February 2024).
